# Regulation of Innate Immune Responses by Autophagy: A Goldmine for Viruses

**DOI:** 10.3389/fimmu.2020.578038

**Published:** 2020-10-06

**Authors:** Baptiste Pradel, Véronique Robert-Hebmann, Lucile Espert

**Affiliations:** IRIM, University of Montpellier, CNRS UMR 9004, Montpellier, France

**Keywords:** autophagy, viruses, innate immunity, co-evolution, ATGs

## Abstract

Autophagy is a lysosomal degradation pathway for intracellular components and is highly conserved across eukaryotes. This process is a key player in innate immunity and its activation has anti-microbial effects by directly targeting pathogens and also by regulating innate immune responses. Autophagy dysfunction is often associated with inflammatory diseases. Many studies have shown that it can also play a role in the control of innate immunity by preventing exacerbated inflammation and its harmful effects toward the host. The arms race between hosts and pathogens has led some viruses to evolve strategies that enable them to benefit from autophagy, either by directly hijacking the autophagy pathway for their life cycle, or by using its regulatory functions in innate immunity. The control of viral replication and spread involves the production of anti-viral cytokines. Controlling the signals that lead to production of these cytokines is a perfect way for viruses to escape from innate immune responses and establish successful infection. Published reports related to this last viral strategy have extensively grown in recent years. In this review we describe several links between autophagy and regulation of innate immune responses and we provide an overview of how viruses exploit these links for their own benefit.

## Introduction

Cellular catabolism is ensured by both the Ubiquitin Proteasome System and the process of autophagy acting in a coordinated manner in order to maintain homeostasis (1). However, some specificities exist between both mechanisms; the Ubiquitin-Proteasome System is mainly responsible for the degradation of short-lived proteins, while autophagy is able to degrade several kinds of substrates, such as long-lived proteins, aggregates, or entire organelles. Among their cellular functions both systems are highly involved in the regulation of innate immune responses upon pathogenic infection.

Upon viral infection, host cells possess an arsenal of innate responses to protect themselves and their neighbors, counteracting pathogen replication and spread. Pattern Recognition Receptors (PRRs) orchestrate the detection of Pathogen-Associated Molecular Patterns (PAMPs), triggering an anti-microbial response by an important shift in transcriptional activity (2). Upon stimulation of PRRs, several transcription factors are activated and translocate to the nucleus. This leads to the transcription and expression of hundreds of genes, particularly pro-inflammatory cytokines, including Interleukin-1;−6;−18 (IL-1; IL-6; IL-18), Tumor Necrosis Factor α (TNF-α), and type I Interferon (type I IFN) (3). These cytokines contribute to the induction of a local inflammatory state and are able to trigger the expression of anti-viral genes, such as Interferon-Stimulated Genes (ISGs) (4).

Autophagy is involved in major biological processes, for instance in development (5), cellular homeostasis (6), and anti-microbial immunity (7). Hence, autophagy is important for several features in physiological settings, and its dysregulation is found in physiopathological conditions (8). Autophagy is described as a major anti-viral mechanism capable of targeting viral components in a process called virophagy (9). Accordingly, viruses have evolved and found ways to inhibit autophagy, but also to use autophagy for their own benefit. Targeting the regulation of the innate immune response by autophagy is an interesting way for viruses to escape from it themselves. Indeed, viruses have been described to have both positive and negative regulatory functions on a growing number of innate immune sensors. It is important to note that these regulatory effects may be linked to autophagy *per se*, but also to autophagy-independent functions of autophagy-related proteins. Thus, even if autophagy is not the only regulatory pathway involved, it is now considered an important regulator of the innate immune response. In this review, we describe the different innate immune signaling pathways that can be counteracted or used by viruses for their own benefit.

## Overview of the Process of Autophagy

**Figure 1:**
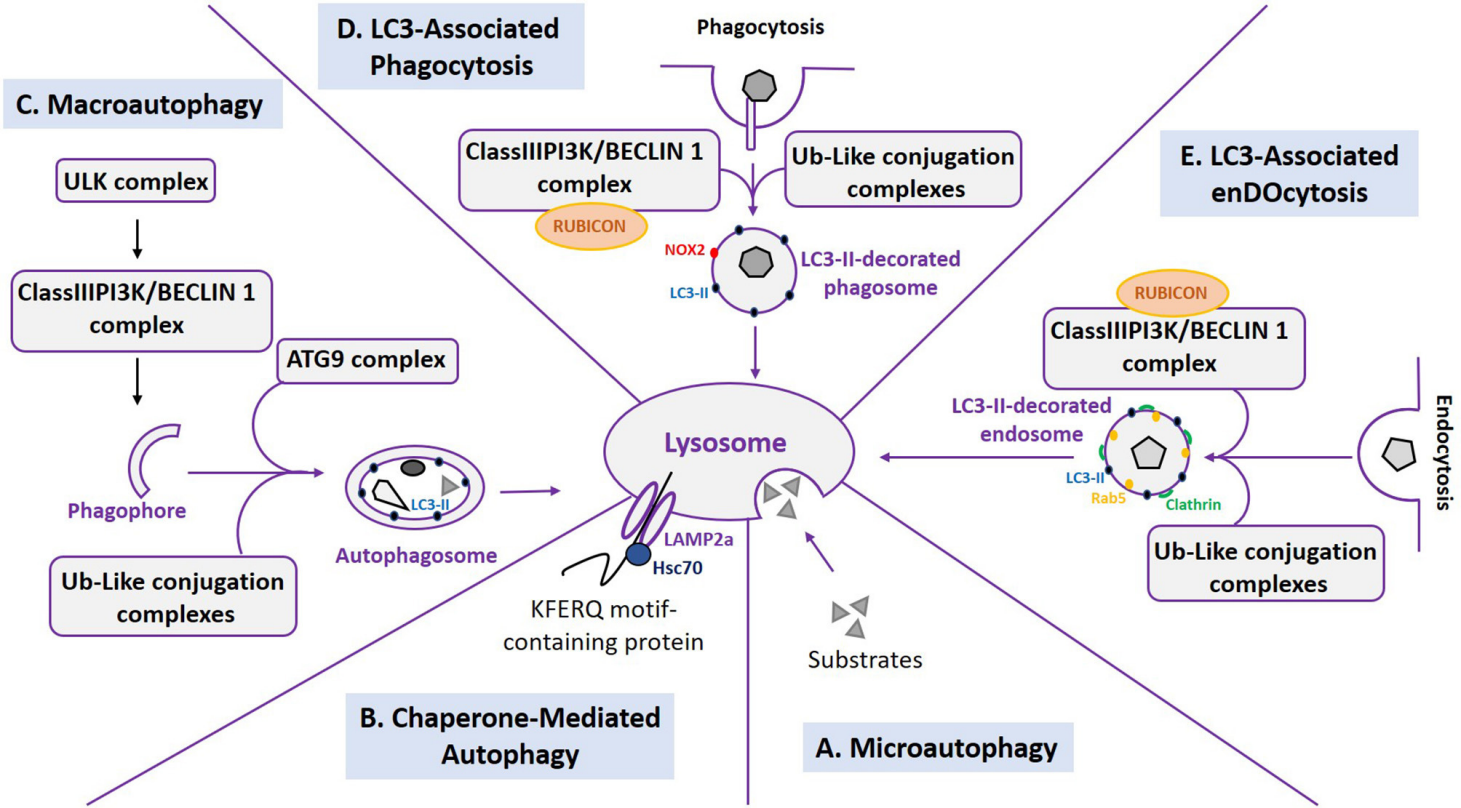
Different processes of autophagy and associated pathways. The lysosome is a central organelle for the processes of autophagy and associated pathways. **(A)** Microautophagy is characterized by the lysosomal degradation of cytosolic components through a direct invagination of lysosomal membranes. **(B)** Chaperone-Mediated Autophagy (CMA) is responsible for the degradation of KFERQ-containing proteins through their interaction with Hsc70 and the lysosomal receptor LAMP2A. **(C)** Macroautophagy is characterized by the formation of double-membrane vesicles (autophagosomes) which ultimately fuse with the lysosome. Several complexes are involved in this process: the ULK complex and the ClassIIIPI3K/BECLIN1 complex for the initiation step, the Ub-like conjugation complexes, and the ATG9 complex for the elongation step. **(D)** LC3-associated Phagocytosis (LAP) shares signaling complexes with macroautophagy and leads to the degradation of phagocytosed components. **(E)** LC3-associated endocytosis (LANDO), similar to LAP, shares signaling complexes with macroautophagy and leads to the degradation of endocytosed components.

The term autophagy refers to “self-eating” and is the lysosomal degradation of intracellular components. It includes three distinct mechanisms: microautophagy, Chaperone Mediated-Autophagy (CMA), and macroautophagy (Figure 1).

Microautophagy is a process involving the protrusion or invagination of lysosomal membranes, leading to engulfment of cytosolic components directly into the lysosome for their subsequent degradation (Figure 1A) (10). CMA is responsible for the degradation of KFERQ-containing proteins (11). This peptide motif is required for binding to the Heat shock cognate 71 kDA protein (Hsc70) (12), a cytosolic chaperone that delivers these protein into the lysosome via its interaction with Lysosome-Associated Membrane Protein type 2A (LAMP2A) (Figure 1B) (13). Despite increasing knowledge over recent years on these two mechanisms, the most well-characterized process of autophagy remains macroautophagy, thus hereafter referred to as autophagy.

Historically deciphered in yeast models, autophagy involves more than 40 AuTophaGy-related (ATG) genes. Eighteen of them are highly conserved among eukaryotes and comprise the core autophagy machinery (14–16). Briefly, autophagy is characterized by the formation of double-membrane vesicles that engulf cytoplasmic portions and ultimately fuse with lysosomes responsible for the degradation of sequestered material (Figure 1C). The process can be delineated into three distinct steps:

(i) The initiation step: This is dependent on two main complexes: the uncoordinated-51-like protein kinase (ULK) complex. This is composed of ULK1 or ULK2, ATG13, Focal adhesion kinase family Interacting Protein 200 (FIP200), and ATG101 (17). The second complex is the class III phosphatidylinositol 3-kinase (PtdIns3K)/BECLIN 1 (BECN1) complex composed of Vacuolar Protein Sorting 34 (VPS34), VPS15, ATG14, Nuclear Binding Receptor Factor-2 (NBRF2), Activating-Molecule in Beclin-1 Regulated Autophagy protein (AMBRA1), and BECN1. Upon induction of autophagy, the ULK1 complex is able to activate the class III PtdIns3K/BECN1 complex, leading to Phosphatidylinositol-3-phosphate (PI3P) production; this is an important lipid for the subsequent recruitment of other ATGs and the formation of a cup-shaped membrane called the phagophore (18). The origin of the phagophore membrane can depend on various factors, such as cell type and physiological conditions (19). There has been reports so far on the implication of the endoplasmic reticulum (20), Golgi apparatus (21), plasma membranes, and mitochondria via ER contact sites (22, 23) and the ER-Golgi Intermediate Compartment (ERGIC) (24). Among the ATG proteins, ATG9A is the only transmembrane protein within the autophagy core machinery and it is essential for the initiation of autophagosome formation. Even if its precise role in autophagy remains elusive, it is thought to act by supplying components, such as proteins and lipids, to the autophagosomal membranes (15, 25).
(ii) The elongation step: This corresponds to phagophore expansion, in which two sophisticated ubiquitination (Ub)-like conjugation complexes are involved. The first one is responsible for the conjugation between ATG5 and ATG12, mediated by the E1-activating enzyme ATG7 and the E2-conjugating enzyme ATG10. Then, ATG16L1 binds to ATG5-ATG12 conjugates in a non-covalent manner (26). The second conjugation system involves ATG7, then the E2-conjugating enzyme ATG3, and finally the ATG5-ATG12-ATG16L1 complex. This behaves as an E3 ligase enzyme for the conjugation of ATG8 to a lipid called a Phosphatidylethanolamine (PE). The ATG8 family is composed of seven homologs: Microtubule-Associated Protein 1 Light Chain 3 A, B1, B2, C (MAPLC3A, MAPLC3B1, MAPLC3B2, MAPLC3C), Gamma-AminoButyric Acid Receptor-Associated Protein (GABARAP), and GABARAP-like 1, 2 (GABARAPL1, GABARAPL2). These are subjected to an ATG4 cleavage before conjugation to PE (26). ATG8-PE conjugates are anchored onto elongating autophagic membranes, leading to the formation of a closed double-membrane vesicle called the autophagosome that engulfs its substrates (26).
(iii) The maturation step: This consists in the fusion between the autophagosome and the lysosomal compartment. This step is achieved by the UV Resistance-Associated Gene (UVRAG)-containing class III PtdIns3K complex. Many other actors are also involved, such as the Rab7 GTPase and the soluble N-ethylmaleimide-sensitive factor attachment protein receptor (SNARE) complex. This is composed of Syntaxin 17 (STX17), SyNaptosomal-Associated Protein 29 (SNAP29), and Vesicle-Associated Membrane Protein 8 (VAMP8) (27).

Fusion leads to the formation of a structure called the autolysosome, in which the degradation of the sequestered materials occurs by lysosomal hydrolases. It is noteworthy that the ATG8-PE conjugate is present on autophagosomes throughout the entire process of autophagy and is degraded in the lysosome, making it a good marker for monitoring autophagic flux. Finally, resulting metabolites are transported into the cytosol in a recycling step (28).

Although initially considered a random mechanism, it is now well-established that autophagy can be highly specific through the action of selective autophagy receptors (SARs) (29). These proteins are able to target highly variable yet specific cargos and recruit the autophagy machinery for their degradation (30). For instance, autophagy can selectively degrade aggregated proteins, damaged organelles (e.g., mitochondria or peroxisomes), viral proteins, and even entire intracellular pathogens. Mitophagy is one of the most well-described and selective processes of autophagy. For example, one mitophagy pathway involves the PTEN-Induced Kinase/Parkin E3 ubiquitin ligase (PINK/PARKIN) protein pair. This pathway starts with the accumulation of PINK1 on damaged mitochondrial outer membranes. This leads to the recruitment and the activation of PARKIN and the massive ubiquitination of several mitochondrial outer membrane proteins. These ubiquitinated proteins are recognized by autophagy receptors, triggering the engulfment of mitochondria in autophagosomes (31).

SARs are able to bind substrates and lead them to the expanding phagophore. On one hand, substrates are often ubiquitinated and therefore recognized by the ubiquitin-binding domain present on most SARs. On the other hand, SARs contain an LC3-Interacting Region which allows the targeting of selected substrates to phagophores (30). Many SARs have been identified in mammals (32). The p62/SQSTM1-Like Receptors (SLRs), which are the most studied SAR family, include p62/SeQueSTosoMe1 (SQSTM1), Neighbor of BRCA1 gene 1 protein (NBR1), OPTiNeurin (OPTN), Nuclear Dot Protein 52 (NDP52), Tax1-Binding Protein 1 (TAX1BP1), and Coupling of ubiquitin conjugation to ER degradation protein 5 (Cue5). All these SLRs have their own selective cargo (33). In addition, there are other proteins involved in selective autophagy, such as NIP-3 like protein X (NIX) in mitophagy (34). More recently, the TRIpartite Motif (TRIM) proteins have been linked to autophagy. For the vast majority, TRIMs are multi-functional proteins that contain an N-Terminal RING-finger domain that acts as an E3 ubiquitin ligase. They also contain one or two zinc finger domains, B1 and B2 boxes, and finally a coiled-coiled domain (35). Recently, several TRIM proteins have been described as both SARs and autophagy platforms, enabling the assembly of the ULK1 complex, in turn describing a new process called “precision autophagy” (36).

ATG8 proteins, hereafter referred to as LC3 (e.g., MAP1LC3B1), are present on autophagic membranes from the elongation to the degradation step in their conjugated form (LC3-PE known as LC3-II). Therefore, monitoring LC3-II protein expression is a classic approach in studying autophagic flux (37). However, LC3 is also involved in non-canonical autophagy. It is now well-established that components of autophagy can intersect with the phagocytosis pathway in a process called LC3-Associated Phagocytosis (LAP) (Figure 1D) (38). The anchoring of LC3 on phagosomes is thought to favor phagosome fusion with lysosomes, even if the role of LC3 is still debated. Many others ATGs are implicated in this pathway, though RUn domain BECN1-Interacting and cysteine-rich domain-CONtaining protein (RUBICON) is indispensable. This protein is a negative regulator of autophagy and behaves as a LAP activator. The class III PtdIns3K/BECN1 and ATG5-ATG12 complexes are also involved in LAP but not the ULK1/2 complex (39). Recently, LC3 has been found associated with Rab5 and Clathrin-positive endosomes. This new process is called LC3-Associated eNDOcytosis (LANDO) (40). Similar to LAP, LANDO requires Rubicon and ATG5 activity but not FIP200. Interestingly, a recent study has shown the ability of LC3 to be conjugated to another lipid, phosphatidylserine, expanding the possible roles of non-canonical autophagy pathways (41). Further studies are required to understand the role and the mechanism of these processes.

## Modulation of Antiviral Innate Immunity by Autophagy

**Figure 2:**
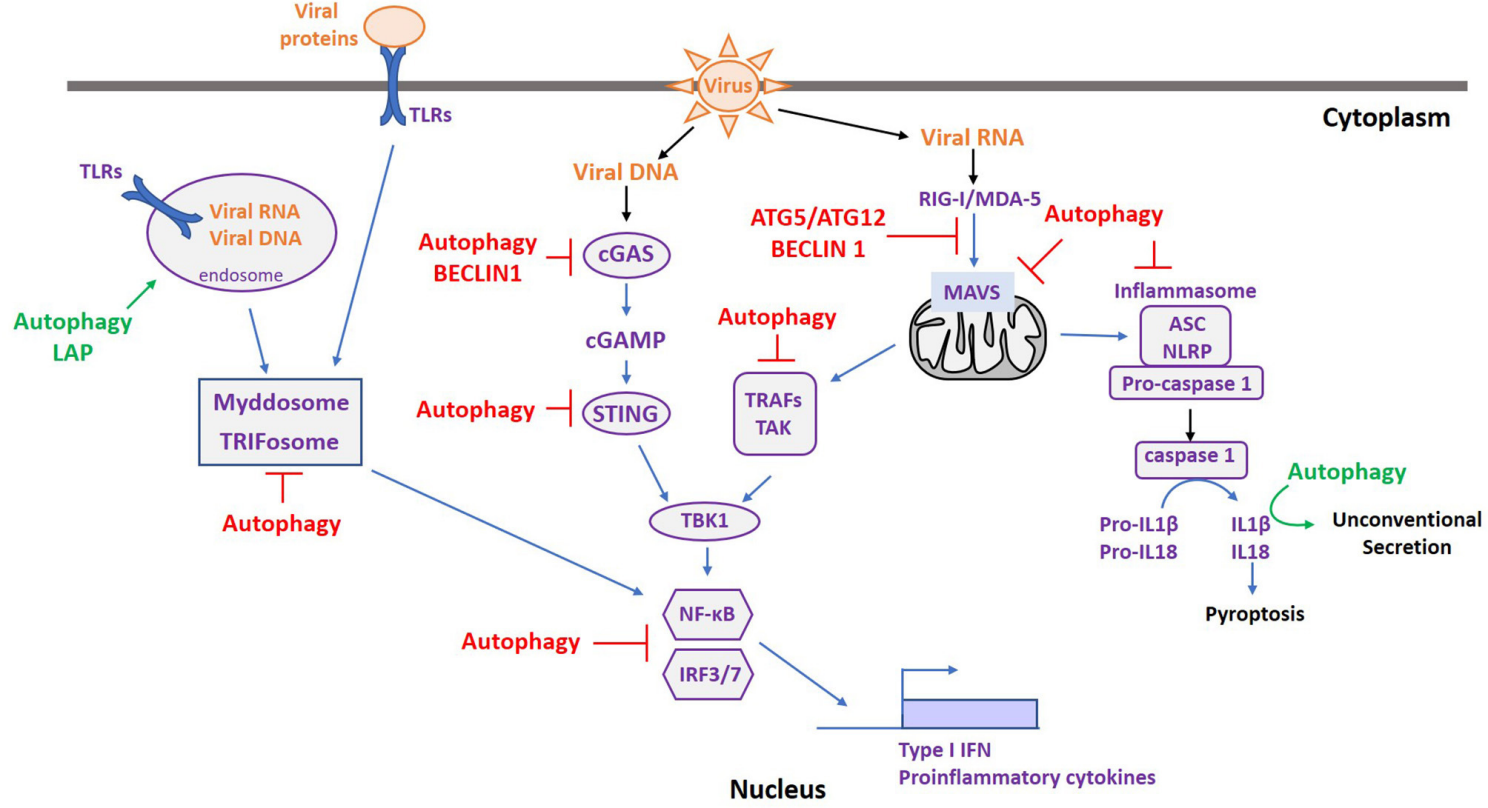
Autophagy and ATGs are Master regulators of immune sensing pathways following pathogen infection. Upon viral infection, various PRRs are activated depending on the type of PAMPs. Autophagy can favor the innate immune response by facilitating PAMP recognition (green), participating in cytokine secretion (green), or by down-regulating the immune response to prevent deleterious effects of prolonged immune activation (red).

Autophagy is an intrinsic pathway of innate immunity. Consequently, autophagy intersects with all the innate immune signaling pathways activated upon viral infection. Indeed, it favors the innate immune response by participating in cytokine secretion and by down-regulating the immune response to prevent deleterious effects of prolonged immune activation. Figure 2 recapitulates the intersections between autophagy and the major innate signaling pathways activated following viral infection. The different signaling mechanisms are detailed below.

### Toll-Like Receptors (TLRs)

TLRs are classified in two subfamilies based on their localization (42): those expressed at the plasma membrane, encompassing TLR1, TLR2, TLR4, TLR5, TLR6, and TLR10, and the intracellular ones expressed on endosomes, including TLR3, TLR7, TLR8, TLR9, TLR11, TLR12, and TLR13. Each TLR interacts with a specific PAMP, and these interactions increase with the formation of TLR homo or heterodimers. TLR3, TLR7, TLR8, and TLR9, which recognize double-stranded RNA (dsRNA) (TLR3), single-stranded RNA (ssRNA) (TLR7/8), and CpG DNA (TLR9), are mainly involved in viral infections (43). In addition, TLR2 and TLR4 contribute to anti-viral immunity by the recognition of viral proteins (44). Following PAMP detection, TLRs transduce a signal through two distinct pathways: (i) the recruitment of a signaling platform called the “Myddosome.” This contains the Myeloid Differentiation Primary Response 88 (Myd88) and IL-1 Receptor-Associated Kinase (IRAK) protein family members (45). Downstream of the Myddosome structure, TNF Receptor-Associated Factor 6 (TRAF6) is activated, triggering the Nuclear Factor-Kappa B (NF-κB) pathway involved in the expression of pro-inflammatory cytokines (45). (ii) The activation of the “Triffosome.” Here intervene Toll/interleukin-1 Receptor domain-containing adaptor protein inducing InterFeron-β (TRIF), TRAF3/6, and Tank-Binding Kinase 1 (TBK1). This induces the nuclear translocation of the Interferon Regulatory Factor 3 (IRF3) transcription factor, leading to the transcription of type I IFN (42).

Autophagy is important for both the regulation and the activation of the TLR/NFκB signaling pathway by TLRs. Indeed, several TLRs are found in endosomes, and therefore the presence of PAMPs in these compartments is crucial for their stimulation. Autophagy plays a key role in this process and it has been implicated in the recognition of Vesicular Stomatis Virus (VSV) (46) and Human Immunodeficiency Virus (HIV) (47) via TLR7 activation. Furthermore, non-canonical autophagy, in particular LAP, seems involved in TLR9 activation in several cell types. Indeed, in mouse plasmacytoid dendritic cells (pDCs) treated with DNA-immunoglobulin complexes, TLR9 trafficking, and activity is dependent on autophagy proteins without the requirement of ULK1 (48). In macrophages, TLR9 stimulation with CpG dinucleotides leads to LC3B and inhibitor of nuclear factor kappa-B (IKK-β) recruitment on TLR9-positive endosomes, in turn allowing the expression of type I IFN (49). However, several studies have also shown a negative regulatory function of autophagy on these signaling pathways. For example, upon TLR stimulation autophagy can be activated in order to decrease pro-inflammatory cytokine expression (50, 51) and several factors involved in TLR signaling are targeted for autophagic degradation. This has been shown for TRIF filaments, formed upon TLR activation, that can be degraded by p62/SQSTM1 and TAX1BP1-mediated selective autophagy (52, 53). Moreover, in Lipopolysaccharide (LPS) or polyIC-treated macrophages, TRIM32 is responsible for the ubiquitination of TRIF, leading to its degradation by TAX1BP1-mediated selective autophagy (54). In addition, NF-κB pathway components can intersect with autophagy downstream of the TLR signaling platform. IKK-β is a target of p62/SQSTM1-mediated selective autophagy via its interaction with S-phase Kinase-associated Protein 2 (SKP2) (55). This autophagic degradation can be induced when SKP2 is ubiquitinated by TRIM21 (56) or when its phosphorylation is inhibited (57). Interestingly, Kenny, the NF-κB Essential MOdulator (NEMO) ortholog in *Drosophila*, acts as a selective autophagy receptor for IKK-β in order to negatively regulate NF-κB responses and prevent constitutive inflammation. However, although NEMO in *Drosophila* contains a LC3-Interacting Region, it is not the case for its mammalian orthologs and they are therefore not involved in IKK-β-selective degradation, showing an evolutionary divergence in NEMO function (58). In addition, p65/RelA, one of the two NF-κB transcription factor subunits, is subject to lysosomal degradation. Indeed, LRRC25 is involved in its p62/SQSTM1-mediated selective autophagic degradation (59), while the KFERQ motif in p65/RelA also allows Hsc70 binding and subsequent CMA-mediated degradation (60). Finally, a recent study has shown that autophagy is also involved in the regulation of a non-canonical NF-κB pathway. It has been demonstrated that in TNFα-prolonged treatment of bone marrow-derived macrophages, the NF-κB p100/p52 dimers are targeted by p62/SQSTM1, leading to their autophagic degradation (61).

### NLRPs and Inflammasomes

Nucleotide-binding oligomerization domain, Leucine-rich Repeat and Pyrin domain-containing (NLRP) receptors are PRRs belonging to the NOD-Like Receptor (NLRs) family. Their stimulation with a specific PAMP leads to the formation of a structure named the “inflammasome.” Upon activation, NLRPs recruit Apoptosis-associated Speck-like protein-containing a CARD (ASC) proteins and mediate their binding with Caspase-1 (CASP1). In turn, the inflammasome triggers the catalytic activity of CASP1, which is able to cleave pro-interleukins, such as pro-IL-1β and pro-IL-18, leading to their release (62). The inflammasome is also involved in a specific type of cell death called “pyroptosis,” in which cytosolic components are released in the extracellular matrix upon plasma membrane rupture contributing to local inflammation (63).

Several NLRs have the ability to form inflammasomes, such as NLRP1, NLRP3, and NLR family CARD domain-containing protein 4 (NLRC4). Some additional sensors have the same ability, including the Absent In Melanoma 2 (AIM2) protein or pyrin (62). During viral infection, inflammasomes are activated by either Microbial-Associated-Molecular-Patterns (MAMPs), such as viral RNA or DNA, but also by Damaged-Associated-Molecular-Patterns (DAMPs), which are released following infection (64). Examples of DAMPs are extracellular ATP produced by damaged cells or mitochondrial DNA released into the cytosol (65).

Regulation of inflammasomes by autophagy is an important process for the control of inflammation given dysfunctional autophagy has been involved in several inflammatory diseases (66). In this context, lupus and rheumatoid arthritis are associated with *Atg5* genetic variants and Crohn's disease is associated with *Atg16l1 and Irgm1* genetic variants. First evidence of a role for autophagy in inflammasome activation was described in Dextran Sulfate Sodium (DSS)-treated mice and LPS-treated macrophages both depleted of ATG16L1 expression. The absence of ATG16L1 led to an increase in inflammasome activation, higher levels of IL1β, and enhanced acute colitis in mice (67). This observation has been confirmed in other mouse inflammation models, including sepsis (68) and uveitis (69).

The regulation of inflammasome activation by autophagy involves several mechanisms. Firstly, inflammasome components can be directly targeted by autophagy. In macrophages, inflammasome activation by poly dA:dT triggers autophagy, which in turn acts as a negative feedback regulation by targeting poly-ubiquitinated ASC to lysosomal degradation (70). Likewise in macrophages, TRIM20 can act as an autophagy receptor for NLRP1, NRLP3, and pro-caspase 1, and TRIM21 for the phosphorylated form of IRF3 (71). In the same cell type, TRIM11 can additionally interact with the AIM2 inflammasome and this leads to its p62/SQSTM1-mediated selective degradation (72). Lastly, recent work has shown a role of IRGM1, another susceptibility factor for Crohn's disease, in inflammasome control by autophagy. IRGM1 is able to directly bind to NLRP3 and ASC which prevents their oligomerization. This process is essential for their activation and subsequently leads to their autophagic degradation (73).

Secondly, autophagy can selectively degrade DAMPs involved in inflammasome activation, therefore reducing pro-inflammatory cytokine production. In this process, mitophagy is an important mechanism. Indeed, damaged mitochondria are potent inducers of inflammation through mitochondrial ROS or mitochondrial DNA release into the cytosol. The disruption of autophagy leads to an accumulation of damaged mitochondria, increasing inflammasome activation and therefore pro-inflammatory cytokine production (74). In contrast, in mouse colitis the induction of mitophagy can reduce NLRP3 activation in order to limit inflammation (75). Other studies have suggested a role of PINK/PARKIN-mediated mitophagy in inflammasome activation. For example, depletion of PINK/PARKIN proteins leads to the accumulation of damaged mitochondria and increased inflammasome activation in a mouse model of sepsis (76). It is important to note that mitochondrial quality control by autophagy can occur in a negative retro-control of PRR activation. Indeed, upon TLR2 or TLR4 stimulation, the serine protease Plasminogen Activator Inhibitor-2 (PAI-2) induces autophagy and reduces mitochondrial ROS (77).

### RIG-I-Like Receptors (RLRs)

The sensing of foreign cytosolic RNA is mediated by RLRs. Retinoic acid-Inducible Gene-I (RIG-I) and Melanoma Differentiation-Associated 5 (MDA-5) are the two mains sensors involved in this recognition. They are structurally composed of a DExD/H-box helicase and a C-terminal domain, both important for RNA recognition (78). RIG-I recognizes ssRNA or dsRNA exposing a 5′ di-or-triphosphate moiety found in both positive and negative ssRNA viruses (79). MDA-5 binds long dsRNA and is mainly involved in positive ssRNA and dsRNA viral infections (80). Upon RNA binding, RLRs undergo conformational changes that expose their Caspase Activation and Recruitment Domains (CARDs), crucial for the activation of downstream signaling pathways. Afterwards, RLRs translocate to the mitochondrial membrane and bind to the Mitochondrial AntiViral-Signaling (MAVS) adaptor protein by CARD-CARD interactions. This allows the recruitment of TRAF proteins, triggering TBK1 kinase activity, and leading to the activation of several transcription factors, including IRF3 and IRF7 (78). MAVS is also found on peroxisomes, where RIG-I interaction with peroxisomal MAVS activates IRF1, leading to the production of type III IFN or ISGs (81).

The first report demonstrating crosstalk between components of autophagy and the RIG-I/MAVS signaling pathway involved the ATG5-ATG12 complex independently of autophagy. This complex is able to prevent RIG-I/MAVS association by binding to their CARD domains, resulting in the inhibition of their activation (82). BECN1 is also able to block the interaction between RIG-I and MAVS (83). Furthermore, RIG-I can be negatively regulated by its conjugation with ISG15. This negative feedback is mediated by the proteasomal degradation of ISG-15-RIG-I conjugates (84). However, recent investigations show that autophagic degradation implicating Leucine-Rich Repeat containing (LRRC) proteins is also involved. While LRRC25 binds ISG15-RIG-I conjugates, targeting them for p62/SQSTM1-mediated autophagic degradation (85), LRRC59 prevents the interaction of LRRC25 with ISG15-RIG-I and therefore promotes type I IFN production (86). Following infection, MAVS activation is dependent on its aggregation. In uninfected conditions, the expression of truncated forms of MAVS prevents the aberrant aggregation of full-length MAVS. In the absence of these truncated forms, the autophagy receptor NIX is able to target aberrant MAVS aggregates for autophagic degradation (87). Type I IFN treatment can also lead to MAVS degradation. Indeed, the IGS Tetherin has been shown to induce MAVS ubiquitination via the E3 ubiquitin ligase MARCH8, leading to NDP52-mediated autophagic degradation (88). Moreover, MAVS can be found on mitochondria, resulting in autophagy-regulated mitochondrial dynamics having a crucial role in its activation. The mitochondrial fusion protein mitofusin has been described as important for MAVS aggregation and activation (89), and as a result also for type I IFN production (90). In opposition, mitochondrial fragmentation and mitophagy are able to disrupt MAVS signaling (91, 92).

To conclude, a recent study has shown that the RIG-I pathway induction is able to induce autophagy in several cell types. Indeed, upon RIG-I activation, BECN1 interacts with TRAF6 located on mitochondria and undergoes a K63-polyubiquitination that triggers autophagy. This process could reveal a negative feedback regulation of RIG-I/MAVS activation (93), however further studies are required to understand the role of this process.

### Cytosolic DNA Recognition by the cGAS/STING Pathway

DNA is normally found in the nucleus or mitochondria of eukaryotes. Therefore, the presence of DNA in the cytosol manifests either microbial infection or cell damage. One of the main cytosolic DNA detection pathways depends on the cyclic GMP–AMP Synthase (cGAS) sensor. DNA binding of cGAS triggers conformational changes and activates its enzymatic activity (94). cGAS catalyzes the conversion of GTP and ATP into a dinucleotide second messenger: cyclic GMP-AMP (cGAMP) (95). Afterwards, cGAMP binds to the ER-located adaptor STimulator of INterferon Genes (STING) (96), which undergoes conformational changes and traffics from the ER to the ERGIC, and finally to the Golgi apparatus. Once located in the Golgi apparatus, STING binds to and activates TBK1. Phosphorylated STING acts as a “dock,” recruiting IRF3 which is subsequently phosphorylated by TBK1 (97). Phosphorylated IRF3 dimerizes and translocates to the nucleus where it induces the transcription of type I IFN.

Activation of the cGAS-STING signaling pathway leads to relocation of STING from the ER to the ERGIC and Golgi apparatus. Components of autophagy are crucial to prevent its aberrant activation. Indeed, STING is found on single-membrane ATG9 and LC3B-positives vesicles after double-stranded DNA treatment and this location is essential for the regulation of its activity (98). Besides, STING has been shown to be a target for autophagic degradation in several studies. It has been found that dinucleotides are able to induce autophagy via the dephosphorylation of ULK1, which switches on its kinase activity. ULK1 is then responsible for STING phosphorylation on serine 366, leading to autophagic degradation of STING (99). Another study also identified the p62/SQSTM1 autophagy receptor in STING degradation (100). Autophagy is also able to regulate cGAS. Its interaction with BECN1 leads to a dampened production of cGAMP (101) and p62/SQSTM1 has been shown to bind cGAS, leading to its autophagic degradation (102).

Additional regulatory pathways can be found in autophagic degradation of cytosolic DNA. Accumulated damaged DNA in *Dnase II-*depleted cells is directed to lysosomal degradation preventing STING activation (103). In addition, disrupted mitophagy in PARKIN-depleted mice leads to accumulation of cytosolic release of mitochondrial DNA, triggering pro-inflammatory cytokine release in a STING-dependent manner (104). Finally, recent studies have shown a negative feedback loop involving STING-dependent activation of autophagy. Upon DNA stimulation, activated STING triggers a non-canonical autophagy, which is dependent on ATG5 but not ULK1. This autophagic process is shown to lead STING to either degradation (105) or DNA clearance (106).

### Other Intersections

Autophagy has been shown to interplay with other components of the innate immune response. For example, the phosphorylated form of IRF1 can be degraded by a p62/SQSTM1-mediated autophagy pathway in macrophages, inhibiting pro-inflammatory cytokine production (107). More interestingly, autophagy has been shown to have a direct effect on IL1β secretion, representing an unconventional secretory pathway (108). Autophagy can also dampen cytokine signaling by degrading their receptors, as shown for the TNF-α (109) and IFNα/β receptors (110).

## Viral Manipulation of Autophagy-Dependent Regulation of Innate Immunity

The co-evolution of viruses with their hosts has resulted in the development of various viral strategies to hijack autophagy during innate immune response control. The Table 1 recapitulates the different viral manipulations of autophagy to dampen antiviral innate immune responses.

**Table 1:** Viral manipulation of autophagy-regulated anti-viral innate immune responses.

**Viruses**	**Mechanisms of autophagy manipulation**	**References**
**TLR AND NF-κB SIGNALING PATHWAY**		
VSV, SV5, HIV-1	Autophagy is crucial for TLR7 activation in pDCs	(46, 47, 111)
Enterovirus 71 (EV71); Coxsackievirus (CA16)	Autophagy diminishes TLR7 signaling	(112)
MCMV; HSV-1	The MCMV M45 protein induces NEMO and RIPK1 aggregation and targeting for autophagy through a conserved IPAM motif	(113, 114)
HCV	p62/SQSTM1-mediated selective autophagy targets TRAF6 which dampens the innate immune response	(115)
**INFLAMMASOME SIGNALING PATHWAY**		
IAV	IAV recognition by NOD receptors induces mitophagy which lowers inflammasome activation	(116)
HIV-1	Mitophagy is induced in productive-infected astrocytes which reduces inflammasome activation	(117)
**RIG-I/MAVS SIGNALING PATHWAY**		
VSV	The ATG5-ATG12 conjugate interacts with RIG-I and MAVS CARD domains which prevents their association and their signaling	(82)
VSV	RNF34 ubiquitinates MAVS which leads to its NDP52-mediated selective degradation by autophagy	(118)
CSFV	The NS5A viral protein triggers the BECN1/MAVS interaction which dampens type I IFN production	(119)
HRV16	Trehalose-induced autophagy triggers the ATG5-MAVS-RIG-I interaction which reduces IFNλ production	(120)
JEV	Inhibiting autophagy increases MAVS aggregation and innate responses	(121)
MeV (EDM strain)	Mitophagy is induced in infected cells which downregulates the RIG-I/MAVS signaling pathway and type I IFN production	(92)
EBV	The BHRF1 viral protein induces mitophagy which dampens the MAVS signaling pathway	(122)
IAV	The M2 viral protein inhibits autophagy and interacts with MAVS which triggers innate immune responses	(123)
IAV	The PB1-F2 viral protein induces mitophagy which leads to MAVS degradation and reduced type I IFN production	(124)
Picornaviruses	The 3A viral protein induces G3BP1 degradation via autophagy by increasing LRRC25 expression which leads to reduced levels of type I IFN	(125)
SeV; VSV	RUBICON binds to CARD9 preventing its interaction with 14-3-3 scaffolding proteins which dampens pro-inflammatory cytokine production	(126)
**cGAS/STING SIGNALING PATHWAY**		
HSV-1; MCMV	p62/SQSTM1 targets STING for autophagic degradation	(100)
HSV-1	The activation of STING triggers ATG5-dependent non-canonical autophagy which leads to STING degradation	(105)
HSV-1	STING is targeted by CMA	(127)
HPV-16	The E7 viral protein triggers NLRX1-mediated autophagic degradation of STING	(128)
Betacoronaviruses	PLP2-TM viral proteins induce the interaction of BECN1 with STING which leads to reduced type I IFN production	(129)
Dengue	The NS2B protease cofactor binds cGAS and mediates its autophagic degradation	(130)
EBV	The BHRF1 viral protein induces mitophagy which dampens the STING-signaling pathway	(122)
**OTHERS**		
VSV; IAV (H1N1 strain)	RUBICON binds to IRF3 which leads to decreased production of type I IFN	(131)
HBV	The BPLF1 viral protein induces the formation of TRIM25 aggregates which are degraded by p62/SQSTM1, decreasing activation of IRF3	(132)
HSV-1; PRV	The UL50 viral protein triggers IFNAR lysosomal degradation	(133)
HCV	IFNAR is degraded by autophagy	(110)
TMV	Autophagic degradation of RNA-silencing pathway components: SGS3 and RDR6	(134)
RSV	The NSvc4 viral protein triggers remorin lysosomal degradation	(135)
SeV	NDP52 targets ubiquitinated IRF3	(136)

### Manipulation of the Autophagy-Regulated TLR Signaling Pathway

We previously mentioned that viral infections can trigger an autophagy-dependent activation of TLRs. In VSV (46), HIV-1 (47), and Paramyxovirus Simian Virus 5 (SV5) (111) infection of pDCs, autophagy is crucial for TLR7 activation and the subsequent production of antiviral cytokines. In contrast, TLR activation can be negatively regulated by viral infection-induced autophagy. For example, in a bronchial epithelial cell line infected by the Enterovirus 71 (EV71) and the Coxsackievirus (CA16), autophagy is shown to decrease TLR7 activation. This is associated with a decreased type I IFN production (112). Moreover, viral proteins are able to drive actors of the TLR signaling pathway toward autophagic degradation. This is the case for the M45 protein from Murine CytoMegaloVirus (MCMV), which is shown to bind NEMO and target it for autophagic degradation (113). Another interesting study shows that M45 is in fact able to induce NEMO and Receptor-Interacting serine/threonine Protein Kinase-1 (RIPK1) aggregation; RIPK1 being a key player in necroptosis. Following aggregation, M45 is able to recruit an LC3-Interacting Region-containing protein TBC1D5, which allows the autophagic degradation of induced aggregates. Moreover, ICP6 of Herpes Simplex Virus (HSV) type I is also able to induce RIPK1 aggregation and degradation. It is noteworthy that the aggregation process is dependent on the presence of a peptide motif called Induce Protein Aggregation Motif (IPAM) in these viral proteins. IPAM-containing proteins are also conserved in other herpesviruses (114), suggesting that several viruses of this family can trigger the aggregation of host proteins and their degradation by autophagy. Finally, TRAF6 degradation by p62/SQSTM1-mediated autophagy can decrease innate responses during late stages of Hepatitis C Virus (HCV) infection. This mechanism could play a role in establishing persistent HCV infection (115).

### Manipulation of the Autophagy-Regulated Inflammasome Pathway

The interplay between inflammasomes and autophagy during viral infection relies mainly on mitophagy. Several viruses are able to induce mitophagy to moderate innate immunity and avoid chronic activation of the immune system. Hence, disrupting autophagy or mitophagy can affect the replication of viral RNA and DNA, of which is the case for HCV (137), Transmissible GastroEnteritis Virus (TGEV) (138), Newcastle Disease Virus (NDV) (139), Human ParaInfluenza Virus type 3 (HPIV3) (140), and Hepatitis B Virus (HBV) (141). First evidence of mitophagy regulation of the inflammasome during viral infection was obtained in the context of Influenza A Virus (IAV) infection. Indeed, IAV recognition by the NOD receptor induces mitophagy, therefore restraining inflammasome activation (116). During HIV-1 infection, abortive infection of astrocytes or infection of microglial cells triggers inflammasome activation (142). However, in productive infection of astrocytes, mitophagy is induced and regulates this process (117). Lastly, in PARKIN-depleted mice, VSV infection leads to increased activation of NLRP3 and consequently higher pro-inflammatory cytokine levels. In this context, VSV replication is dramatically reduced and is accompanied with a higher survival rate of mice (143).

### Manipulation of the Autophagy-Regulated RIG-I/MAVS Signaling Pathway

MAVS are often targeted by viruses in order to counteract their associated innate responses. Some viral proteins have been shown to interact with MAVS proteins and dampen their activity. Viral proteases can also cleave MAVS (144). Recently, miR-22 was found to inhibit MAVS expression at the RNA level (145). Viral-induced autophagy has been shown to target MAVS. For example, in VSV-infected cells, the interaction of the ATG5-ATG12 conjugate with RIG-I and MAVS CARD domains has been shown to reduce their downstream signaling pathways (82). Additionally, RING Finger protein 34 (RNF34) induces MAVS ubiquitination leading to its NDP52-mediated autophagic degradation (118). The Classical Swine Fever Virus (CSFV) NS5A protein is able to induce a BECN1/MAVS interaction that reduces type I IFN production (119). Likewise, Trehalose, a molecule constituted of glucose, has a pro-viral role in Human RhinoVirus 16 (HRV-16)-infected primary airway cells. Indeed, by inducing autophagy this component is responsible for the interaction between ATG5 and RIG/MAVS that reduces IFNλ production (120). In Japanese Encephalitis Virus (JEV) infected-cells, inhibiting autophagy is associated with higher levels of MAVS aggregates and thus pro-inflammatory cytokines (121).

MAVS, being a mitochondria-associated protein, is sensitive to mitochondrial dynamics and as a result to mitophagy. The measles virus Edmonson strain viral vaccine is shown to induce p62/SQSTM1-mediated mitophagy that leads to the downregulation of RIG-I/MAVS-dependent type I IFN production (92). In addition, the Epstein-Barr Virus (EBV) encodes BHRF1, a B-Cell Lymphoma-2 (Bcl-2) viral homolog. This is able to induce mitochondrial fission and mitophagy, leading to reduced type I IFN expression via signaling pathway activation, including that of MAVS (122). IAV infection is interesting given IAV expresses two viral proteins harboring opposite effects. On one hand, the M2 protein is a potent inhibitor of autophagy and promotes innate responses by inducing mitochondrial ROS and interacting with MAVS (123). On the other hand, the PB1-F2 protein is able to bind to the mitochondrial protein Elongation factor TU (TUFM) known to induce mitophagy by an interaction with the ATG5-ATG12 conjugate. In this context, PB1-F2-mediated mitophagy leads to MAVS degradation and reduced production of type I IFN (124).

Autophagy can act upstream of the RIG-I/MAVS complex. Indeed, the 3A proteins of several picornaviruses induce LRRC25 expression. LRRC25 binds and induces the Ras-GTPase-activating protein (SH3 domain) binding protein 1 (G3BP) degradation by autophagy. A recent study established that picornaviruses dampen type I IFN production (125) by inducing autophagic degradation of G3BP1 involved in RIG-I signaling (146). Furthermore, RUBICON is able to reduce RIG-I signaling in Sendai virus and VSV infections independently of its regulatory role in autophagy. Mechanistically, RUBICON binds to CARD9, preventing it binding to 14-3-3 proteins. Given 14-3-3 protein interaction with CARD9 is a positive regulator of RIG-I signaling, RUBICON plays an important role in its regulation (126).

### Manipulation of the Autophagy-Regulated cGAS/STING Signaling Pathway

Many studies on STING regulation by autophagy have been carried out in the HSV-1 infection model. Correspondingly, in infected macrophages STING is targeted to p62/SQSTM1-mediated autophagic degradation, a process responsible for decreasing the levels of pro-inflammatory cytokines (100). This degradation is also observed in MEF cells. Here, ATG5-dependent non-canonical autophagy is responsible for the negative feedback of STING activation by degrading STING itself (105). CMA also targets STING during late infection stages in HEK293T cells. This is due to the presence of the KFERQ motif in STING, allowing its interaction with Hsc70 required for CMA targeting (127). Other viruses are also able to block STING activity. For example, the E7 protein from the oncogenic Human Papilloma Virus (HPV) 16 induces NLRX1-mediated autophagic degradation of STING (128). It is worth noting that this event is not linked to a pro-viral but to a pro-tumoral effect. Indeed, abolishment of STING-mediated production of type I IFN leads to decreased T-cell infiltration in head and neck squamous cell carcinoma and therefore contributes to immune escape in tumors.

PLP2-TM viral proteins from several betacoronaviruses, such as SARS COV-1 and MERS, induce the interaction of STING with BECN1. This complex negatively regulates STING activity and therefore type I IFN production (129). Additional targeting of cGAS by autophagy in Dengue-Virus (DENV)-infected cells, in which the DENV NS2B protease cofactor binds to cGAS, leads to cGAS lysosomal degradation. Therefore, production of type I IFN in DENV-infected cells is dampened since cGAS is no longer able to recognize mitochondrial DNA which is potentially released into the cytosol during DENV infection of HEK293T cells (130). Finally, acting on the RIG-I/MAVS signaling pathway, autophagy can also alter the cGAS/STING pathway response. We previously mentioned that the EBV-encoded BHRF1 protein is able to induce mitophagy, inhibiting MAVS signaling and leading to reduced type I IFN expression. In this compelling work, the authors also showed that BHRF1-induced mitophagy is also able to diminish the STING-signaling pathway (122).

### Other Manipulations of Innate Immune Responses

Other components of innate immune responses are also targeted by autophagy during viral infections. For example, RUBICON is able to bind to IRF3, a process that inhibits its activation and favors the replication of several viruses, such as VSV and the H1N1 strain of IAV (131). During HBV infection, the viral protein BPLF1 is able to target TRIM25 degradation by p62/SQSTM1-mediated autophagy. This results in decreased IRF3 activation and subsequently lower levels of type I IFN production (132). Moreover, UL50 proteins from HSV-1 and PseudoRabies Virus (PRV) induce the lysosomal degradation of the Type I IFN receptor 1 (133). Although the mechanism is not yet deciphered, it also occurs in HCV infection (110). Interestingly, manipulation of autophagy for escaping immune responses is also found among plant viruses. Indeed, the RNA silencing pathway is dampened by the turnip Mosaic virus via autophagic degradation of Supressor of Gene Silencing 3 (SGS3) and RNA-Dependent RNA polymerase 6 (RDR6) (134). Likewise, the rice stripe virus protein NSvc4 induces and mediates lysosomal degradation of the plant immune response component, remorin (135). These findings show that control of immune responses by autophagy is a common mechanism used by viruses to enhance their replication and to escape antiviral machinery. To conclude, a recent study has shown that IRF3 is targeted by NDP52-mediated selective autophagy following sendai virus infection in a virus load-dependent manner. The authors suggest that this process could precisely regulate IRF3 activity and consequently the type I IFN response to viral infection (136).

## Discussion

A constant selective pressure is imposed on viruses by their hosts, leading pathogens to adapt in order to replicate efficiently. Consequently, even if autophagy is known to be an important process in fighting viruses, some viruses have evolved various strategies to exploit it for their own benefit. In recent years, an increasing number of studies have reported a role of autophagy in the regulation of the immune system and how this can be hijacked by pathogens. Interestingly, this hijacking seems to be a common feature between pathogenic viruses independently of their genomic structures or their specific hosts, likely because autophagy is occurring in all eukaryotic cells. We describe in this review the current knowledge still expanding in the field. The description of the regulation of innate immunity by autophagy is often focused on one single innate immune pathway. However, several reports indicate a cross-talk between multiple cellular immune responses (147), showing that autophagy could regulate several different innate immune pathways during the same infection (122, 148). Therefore, the targeting of one innate immune sensor by ATGs or SARs could affect more than just its related pathway. This theory should be taken in consideration in future works.

It is well-known that innate responses can be deleterious by inducing an uncontrolled inflammation in many viral infections, leading to severe symptoms and sometimes death. This is particularly well-illustrated in the case of the SARS-CoV2 infection, responsible for the COVID-19 pandemic. In this context, autophagy could be considered as an important process regulating both viral replication and the innate immune response. Only a few studies have been conducted to study the link between the control of innate responses by autophagy and the outcome of the infection *in vivo*. Many studies remain descriptive and further work is required to decipher the related mechanisms. It would be particularly important to characterize the effect of autophagy-modulating drugs during viral infections. Moreover, the recently discovered LAP and LANDO processes suggest that there is still a great deal to discover with respect to co-evolution between viruses and their hosts.

Humanity is facing the emergence of a large number of new pathogens, including zoonoses, particularly due to climate change and human activity. In this light, we will undoubtedly face many new viral emergences in the future. Therefore, the study of infectious diseases is essential to anticipate these future challenges.

Overall, this review shows that autophagy plays a central role in host/pathogen interactions, in particular in the immune response and its hijacking by viruses. Thus, it seems essential to develop our knowledge in this field in order to be able to uncover new therapeutic strategies that combine effects on both viral replication and on the host immune response to infections.

## Author Contributions

BP, VR-H, and LE have all contributed to the writing of the manuscript. All authors contributed to the article and approved the submitted version.

## Conflict of Interest

The authors declare that the research was conducted in the absence of any commercial or financial relationships that could be construed as a potential conflict of interest.
